# Novel *β*-*N*-acetylglucosaminidases from *Vibrio harveyi 650*: Cloning, expression, enzymatic properties, and subsite identification

**DOI:** 10.1186/1471-2091-11-40

**Published:** 2010-09-29

**Authors:** Wipa Suginta, Duangkamon Chuenark, Mamiko Mizuhara, Tamo Fukamizo

**Affiliations:** 1 Biochemistry-Electrochemistry Research Unit, Schools of Chemistry and Biochemistry, Institute of Science, Suranaree University of Technology, Nakhon Ratchasima 30000, Thailand; 2 Department of Advanced Bioscience, Kinki University, 3327-204 Nakamachi, Nara 631-8505 Japan

## Abstract

**Background:**

Since chitin is a highly abundant natural biopolymer, many attempts have been made to convert this insoluble polysaccharide into commercially valuable products using chitinases and *β*-*N*-acetylglucosaminidases (GlcNAcases). We have previously reported the structure and function of chitinase A from *Vibrio harveyi *650. This study t reports the identification of two GlcNAcases from the same organism and their detailed functional characterization.

**Results:**

The genes encoding two new members of family-20 GlcNAcases were isolated from the genome of *V. harveyi *650, cloned and expressed at a high level in *E. coli*. *Vh*Nag1 has a molecular mass of 89 kDa and an optimum pH of 7.5, whereas *Vh*Nag2 has a molecular mass of 73 kDa and an optimum pH of 7.0. The recombinant GlcNAcases were found to hydrolyze all the natural substrates, *Vh*Nag2 being ten-fold more active than *Vh*Nag1. Product analysis by TLC and quantitative HPLC suggested that *Vh*Nag2 degraded chitooligosaccharides in a sequential manner, its highest activity being with chitotetraose. Kinetic modeling of the enzymic reaction revealed that binding at subsites (-2) and (+4) had unfavorable (positive) binding free energy changes and that the binding pocket of *Vh*Nag2 contains four GlcNAc binding subsites, designated (-1),(+1),(+2), and (+3).

**Conclusions:**

Two novel GlcNAcases were identified as exolytic enzymes that degraded chitin oligosaccharides, releasing GlcNAc as the end product. In living cells, these intracellular enzymes may work after endolytic chitinases to complete chitin degradation. The availability of the two GlcNAcases, together with the previously-reported chitinase A from the same organism, suggests that a systematic development of the chitin-degrading enzymes may provide a valuable tool in commercial chitin bioconversion.

## Background

Chitin is a *β*-1,4-linked homopolymer of *N*-acetylglucosamine (GlcNAc), which is found mainly in the exoskeleton of crustaceans, insects and in the cell walls of fungi. Chitin is one of the most abundant polymers in nature and its degradation derivatives are pharmaceutically valuable. for example, chitoligosaccharides can stimulate the immune system to respond to microbial infections and chitin monomers have been shown to act as anti-aging and anti-tumor agents, as well as to relieve the symptoms of osteoarthritis [1-6]. Complete degradation of chitin requires chitinases (EC 3.2.1.14) and *β*-*N*-acetylglucosaminidases (GlcNAcases) or chitobiases (EC 3.2.1.52), so such enzymes could potentially serve as biocatalysts in the production of chitin derivatives of desired sizes during the recycling of chitin biomass.

As well as functioning in chitin degradation by bacteria, GlcNAcases are also known to be key enzymes in the catabolism of glycoconjugates containing *N*-acetylglucosamine residues [7,8] and mutations of the gene encoding a human GlcNAcase homologue (HexA) cause a fatal genetic lipid storage disorder, known as Tay-Sachs disease [9]. In the CAZy database (http://www.cazy.org), GlcNAcases are classified into glycosyl hydrolases family 3 (GH-3) or family 20 (GH-20), which differ in sequence and mode of enzyme action [10,11]. Family-3 GlcNAcases are thought to act by a standard retaining mechanism involving a covalent glycosyl-enzyme intermediate while family-20 enzymes employ a 'substrate-assisted' mechanism involving the transient formation of an oxazolinium ion intermediate [12-15]. Most of the GlcNAcases described hitherto belong to the GH-20 family. To date, only five bacterial GH-3 GlcNAcases have been characterized, including NagZ or ExoII from *Vibrio furnissii *[16], Nag3A from *Clostridium paraputrificum *M-2 [17], NagA from *Streptomyces thermoviolaceus *[18], and NagA and CbsA from *Thermotoga maritima *and *T. neapolitana *[19].

*Vibrio harveyi*, formerly known as *V. carchariae*, is a Gram-negative marine bacterium that causes luminous *Vibriosis*, a serious disease that affects commercially farmed fish and shellfish species [20,21]. We previously reported isolation of the gene encoding endochitinase A from *Vibrio harveyi *type strain 650 for functional and structural characterization [22,23]. In this study, we employed a homology-based strategy to isolate two GlcNAcase genes from the genome of the same *Vibrio *strain. Sequence analysis suggested that the resultant polypeptides were new members of the GH-20 family. Enzymic properties of the GlcNAcases expressed in *E. coli *were investigated. Their kinetic properties and identification of the subsites in the more active enzyme are discussed in further detail.

## Results and Discussion

### Gene isolation and sequence analysis

**Figure 1:**
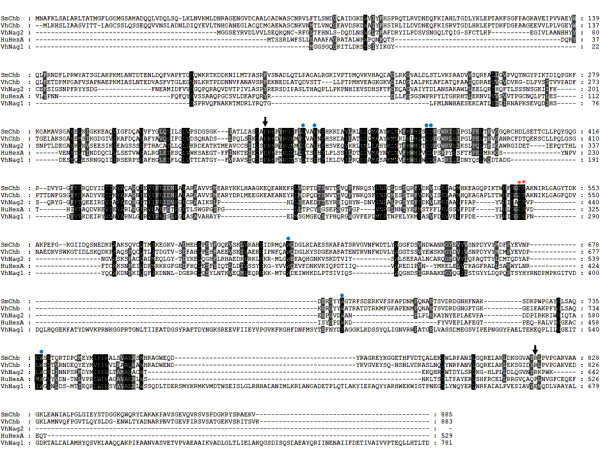
**Amino acid sequence alignment of five GH-20 GlcNAcases**. The sequence alignment was generated by CLUSTAL W and displayed by Genedoc. The amino acid sequence of *S. marcescens *chitobiase: *Sm*Chb (Swiss-Prot: Q54468) was aligned with the corresponding sequence HuHexA: the α-chain of human hexosaminidase (Swiss-Prot: P06865), *V. harveyi *chitobiase: *Vh*Chb (Swiss-Prot: P13670), *V. harveyi *650 *Vh*Nag1(GenBank: HM175715) and *Vh*Nag2 (GenBank: HM175716). The acidic pair important for enzyme catalysis is marked with red asterisks. The residues responsible for diNAG binding are marked with blue dots. Arrows indicate the first and the last residues in the catalytic domain of *Sm*Chb.

The availability of the complete genome sequence allowed us to locate three open reading frames (ORFs), including VIBHAR_03430 (Swiss-Prot: A7MYY8), VIBHAR_06345 (Swiss-Prot: A7N8P3) and, VIBHAR_01265 (Swiss-Prot: A7N1G4) in the genome of *V. harveyi *type strain ATCC BAA-1116 BB120. These reading frames encode uncharacterized proteins with presumed GlcNAcase activity. In an attempt to isolate the genes that encode GlcNAcases in a closely-related organism, three sets of oligonucleotides were designed based on the above-mentioned ORFs. Two homologous DNAs were amplified by the oligonucleotides designed from the VIBHAR_03430 and VIBHAR_01265 ORFs, whereas the DNA fragment compatible with the VIBHAR_06345 ORF could not be amplified successfully. Hence, the first two DNA fragments (hereafter referred to as *VhNag1 *and *VhNag2*) were further cloned and expressed for functional characterization. Nucleotide sequence analysis showed that the *Vh*Nag1 full-length DNA contains 2,343 bp which encode a polypeptide of 88,849 Da, whereas the *Vh*Nag2 full-length DNA contains 1,926 bp, encoding a polypeptide of 73,143 Da. The *p*I values of *Vh*Nag1 and *Vh*Nag2 were calculated to be 4.9 and 5.4, respectively. The nucleotide and corresponding amino acid sequences of the newly-identified GlcNACases have been deposited in the GenBank/EMBL/DDBJ database with assigned accession numbers of HM175715 for *Vh*Nag1 and HM175716 for *Vh*Nag2. Although a BLAST search indicated high sequence similarity of *Vh*Nag1 and *Vh*Nag2 to GlcNAcases from other *Vibrio *species, most of these sequences turned out to be only hypothetical proteins with predicted GlcNAcase activity. Low sequence similarity with all GH-3 members (< 12%) suggested that neither of these enzymes belong to GH-3, but matches of up to 30% with previously studied GH-20 enzymes indicated that they are new GH-20 members. A sequence comparison with eleven functionally-characterized GH-20 enzymes (data not shown) shows that both *Vh*Nag sequences are closest to the α-chain of human GlcNAcase (HuHexA, 30% similarity) [24], followed by *Serratia marcescens *chitobiase (*Sm*Chb, 24%) [25]. Fig. 1 shows a sequence alignment of *Vh*Nag1 and *Vh*Nag2 with three selected GlcNAcases, including *Vh*Chb [26] (the only characterized GlcNAcase from *V. harveyi*), HuHexA and *Sm*Chb (which have the highest sequence similarities to the *Vh*Nag sequences). Since the structural details of *Sm*Chb are well studied, further sequence comparisons were with *Sm*Chb. As seen in Fig. 1, a number of consensus residues are identified, a few of which may participate in the catalytic function of the *Vibrio *GlcNAcases. The most obvious of these are the acidic pairs Asp287-Glu288 (in *Vh*Nag1) or Asp437-Glu438 (in *Vh*Nag2) (Fig. 1, red asterisks) as they are completely aligned with Asp539-Glu540 of *Sm*Chb. These two residues were previously shown to be crucial for the catalytic function of *Sm*Chb, Glu540 acting as the catalytic residue [13,25]. Other residues found to be involved in binding the substrate in the enzyme-substrate complex of *Sm*Chb-diNAG include Asp346, Arg349, Asp378, Asp379, Trp616, Trp685, and Trp737. These residues are completely conserved among the GlcNAcase species (Fig. 1, blue dots).

### Recombinant expression and mass identification

**Figure 2:**
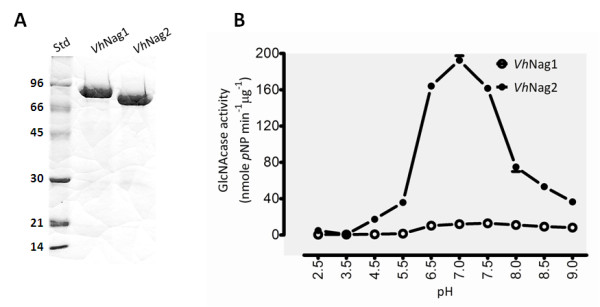
**Physical properties of *V. harveyi *GlcNAcases**. A) SDS-PAGE analysis of *Vh*Nag1 and *Vh*Nag2 expressed in *E. coli *and purified by Ni-NTA agarose chromatography. Std: low molecular weight protein markers. B) pH/activity profiles of *Vh*Nag1 and *Vh*Nag2. GlcNAcase activity was assayed at different pH values from 2.5 to 9.5 at 37°C for 10 min using *p*NP-GlcNAc as substrate

The full-length *Vh*NAg1 and *Vh*Nag2 DNAs were subsequently cloned into the pQE60 expression vector, which provides high-level expression in *E. coli *M15 of *C*-terminally (His)_6_-tagged polypeptides. The overall yields of the purified recombinant proteins obtained after Ni-NTA affinity chromatography were 5 - 10 mg/ml of bacterial culture. Fig. 2A shows SDS-PAGE analysis, displaying a single band of *Vh*Nag1 migrating to slightly below the 96 kDa marker and *Vh*Nag2 closer 66 kDa. The molecular masses estimated from their mobilities are consistent with the theoretical masses of the enzymes (88,849 Da for *Vh*Nag1 and 73,143 Da for *Vh*Nag2). In peptide mass fingerprinting, 9 and 13 *Vh*Nag1 peptides unambiguously matched internal peptides of GlcNAcases from *V. angustum *and *Photobacterium sp*, respectively. For *Vh*Nag2, 20 and 21 peptides were identical with the internal peptides of *V. parahaemolyticus *and *V. alginolyticus *GlcNAcases (see additional file 1, Table S1). This peptide mass identification strongly indicates that the proteins expressed in *E. coli *are GlcNAcases.

### Assessment of GlcNAcase activity and kinetic studies

Both of the purified GlcNAcases were active against *p*NP-GlcNAc, but *Vh*Nag2 was found to be much more active than *Vh*Nag1. We suspect that the full-length *Vh*Nag1 is expressed as a pro-enzyme, which requires proteolytic processing to attain its full activity. The hydrolysis of *p*NP-GlcNAc by *Vh*Nag1 and *Vh*Nag2 was determined as a function of time (additional file 2, Fig. S1). *Vh*Nag2 activity was significantly greater than that of *Vh*Nag1 over the entire course of reaction. In the reaction progress curves, the activity of both enzymes was constant for up to 15 min, and then began to decrease at longer incubation times. Therefore, the reaction time was set to 10 min to ensure that initial velocities were measured in subsequent kinetic experiments.

**Figure 3:**
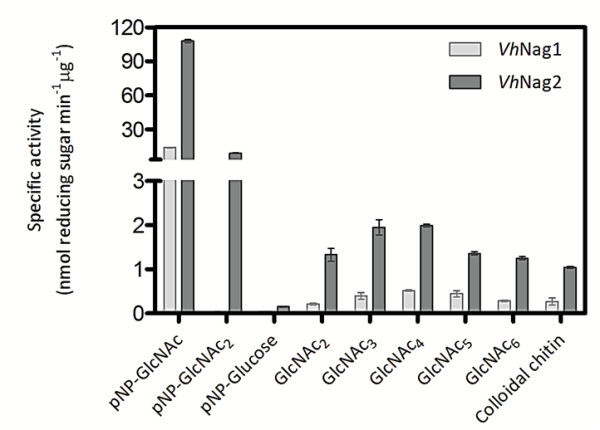
**Specific hydrolyzing activity of *V. harveyi *GlcNAcases**. The enzymic activity of *Vh*Nag1 and *Vh*Nag2 was determined using the colorimetric assay for *p*NP-glycoside substrates and using the reducing sugar assay for chitooligosaccharides substrates. The reactions were set up as described in the text.

The effect of pH on GlcNAcase activity was examined with *p*NP-GlcNAc as substrate. The pH activity profiles of *Vh*Nag1 and *Vh*Nag2 were typical bell-shaped curves as seen for most glycosyl hydrolases. Fig. 2B shows enzymic activity decreasing at low and high pH values. The maximal activity of *Vh*Nag1 was at pH 7.5 and of *Vh*Nag2 at pH 7.0. When specific hydrolytic activity was determined with various substrates (Fig. 3), *p*NP-GlcNAc was found to be the most effective. While *Vh*Nag2 could hydrolyze all the three *p*NP-glycosides (although *p*NP-GlcNAc_2 _and *p*NP-Glucose were respectively hydrolyzed 300-fold and 700-fold less efficiently than *p*NP-GlcNAc), *Vh*Nag1 was only active with *p*NP-GlcNAc. Both enzymes could hydrolyze chitooligosaccharides (GlcNAc_2-6_) and colloidal chitin with *Vh*Nag2 being at least ten-times more active than *Vh*Nag1. Of the natural glycosides, chitotetraose was the best substrate and chitotriose the second best. In contrast, chitobiose, chitopentaose and chitohexaose were only fair substrates, and insoluble (colloidal) chitin was the poorest of all (Fig. 3).

The kinetic parameters of the hydrolytic activity of the two GlcNAcases were further assessed. As shown in Table 1, *k*_cat_/*K*_m _with *p*NP-GlcNAc was 11-fold greater for *Vh*Nag2 than for *Vh*Nag1. It was noted throughout this study that *Vh*Nag1 was tended to undergo rapid and progressive loss of GlcNAcase activity. Although several attempts were made to obtain the kinetic values of this higher-M_*r *_enzyme, the data acquired with *Vh*Nag1 could not be evaluated precisely, and we decided not to present it in this study. In accordance with our earlier demonstration (see Fig. 3), *Vh*Nag2 had the lowest *K*_m _and highest *k*_cat_, yielding overall *k*_cat_/*K*_m _of 4,935 M^-1^s^-1 ^towards *p*NP-GlcNAc. When natural substrates were compared, *Vh*Nag2 had the greatest catalytic activity with GlcNAc_4 _(*k*_cat_/*K*_m _304 M^-1^s^-1^), followed by GlcNAc_3 _(228 M^-1^s^-1^), GlcNAc_5 _(181 M^-1^s^-1^), GlcNAc_6 _(166 M^-1^s^-1^), and GlcNAc_2 _(56 M^-1^s^-1^). Overall, *k*_cat_/*K*_m _of *Vh*Nag2 with *p*NP-GlcNAc is 88-fold greater than with GlcNAc_2 _(the poorest glycoside substrate) and 16-fold greater than with GlcNAc_4 _(the best glycoside substrate). In general, the glycosidic bond of *p*NP-GlcNAc is more easily cleaved than that of GlcNAc-GlcNAc, because of the higher electron-withdrawing capacity of *p*-nitrophenyl moiety. However, the rate of hydrolysis of *p*-nitrophenyl glycoside depends on the affinity to the enzyme used. For example, Keyhani & Roseman [27] showed that *Vibrio furnssii β*-GlcNAcidase (exoI) hydrolyzed *p*NP-GlcNAc about 5-fold faster than GlcNAc_2_, but 1.4 fold slower than GlcNAc_3-6_, whereas Kubota *et al*. [28] reported that *Streptomyces thermoviolacacus *NagC could hydrolyze *p*NP-GlcNAc and GlcNAc_2 _at equal rates. On the other hand, its activity with the *p*NP substrate was between 2- to 3.5-fold greater than with GlcNAc_3-5_.

**Table 1:** Kinetic parameters of chitin oligosaccharide hydrolysis.

Substrate	***K***_**m **_**(μM)**	***k***_**cat **_**(s**^**-1**^**)**	***k***_**cat**_**/*K***_**m **_**(M**^**-1 **^**s**^**-1**^**)**
*p*NP-GlcNAc (*Vh*Nag1)	172 ± 48^a^	0.08	465
*p*NP-GlcNAc(*Vh*Nag2)	77 ± 17	0.38	4,935
GlcNAc_2_	179 ± 52	0.01	56
GlcNAc_3_	441 ± 98	0.10	228
GlcNAc_4_	329 ± 93	0.10	304
GlcNAc_5_	496 ± 78	0.09	181
GlcNAc_6_	421 ± 76	0.07	166

^a ^Results are means ± SD (n = 3 experiments for each substrate).

The initial velocity of the reaction catalyzed by *V. harveyi *GlcNAcases was measured at 37°C, 10 min with varied concentrations of chitin oligosaccharides from 0-500 μM. The release of products was determined by the reducing sugar assay (see text). The kinetic experiments were carried out in triplicate. The kinetic parameters (*K*_m_, *k*_cat_, and *k*_cat_/*K*_m_) were estimated from a non-linear regression function available in GraphPad Prism v.5.0.

### Time course of chitin oligosaccharide hydrolysis by TLC and HPLC

**Figure 4:**
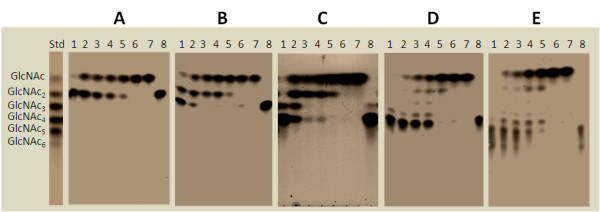
**Time course of chitooligosaccharide hydrolysis by TLC**. A reaction mixture (20 μl), containing 800 ng *Vh*Nag2 and 2.5 mM of A) GlcNAc_2_; B) GlcNAc_3_; C) GlcNAc_4_; D) GlcNAc_5_; or E) GlcNAc_6 _in 0.1 M phosphate buffer, pH 7.0, was incubated at various times at 37°C, and then analyzed by TLC. Sugar products were detected with aniline-diphenylamine reagent. Lanes: std, a standard mix of GlcNAc_1-6_; 1-7, incubation at 1, 5, 10, 15, 30 min, 3 h and 18 h, respectively; and 8, substrate control.

Degradation patterns of chitooligosaccharides by *Vh*Nag2 were analyzed by TLC (Fig. 4). The most significant result obtained from TLC analysis is that *Vh*Nag2 acted exolytically, yielding GlcNAc as the end product from all chitin oligomers. Additionally, GlcNAc_3 _and GlcNAc_4 _were degraded rapidly and almost completely within 10 min (Fig. 4B and 4C), whilst the reaction of GlcNAc_5 _was not complete until 1 h of incubation (Fig. 4D). The degradation of GlcNAc_2 _(Fig. 4A) and GlcNAc_6 _(Fig. 4E) was even slower, complete hydrolysis of these two substrates being attained only after 2 h of incubation.

**Figure 5:**
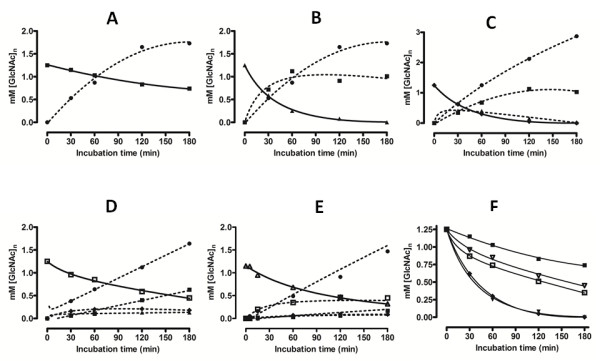
**Product analysis of by quantitive HPLC**. Purified *Vh*Nag2 (50 ng) was added to a reaction mixture containing 5 mM (GlcNAc)_n _in 0.2 M mM phosphate buffer, pH 7.0. The mixture was quenched after the indicated reaction times at 30°C by the addition of 0.1 M NaOH and applied to calibrated HPLC. For each substrate (solid line), the calculated concentrations of the products formed (broken line) are shown. Plots show the hydrolysis of A) (GlcNAc)_2_; B) GlcNAc_3_; C) GlcNAc_4_; D) GlcNAc_5_; E) GlcNAc_6; _and F) the estimated hydrolytic rate of each substrate as a function of time. Substrates and products are represented by (GlcNAc) (black circle); (GlcNAc)_2 _(black square); (GlcNAc)_3 _(black triangle); (GlcNAc)_4 _(black diamond); (GlcNAc)_5 _(white square); and (GlcNAc)_6 _(white triangle).

**Figure 6:**
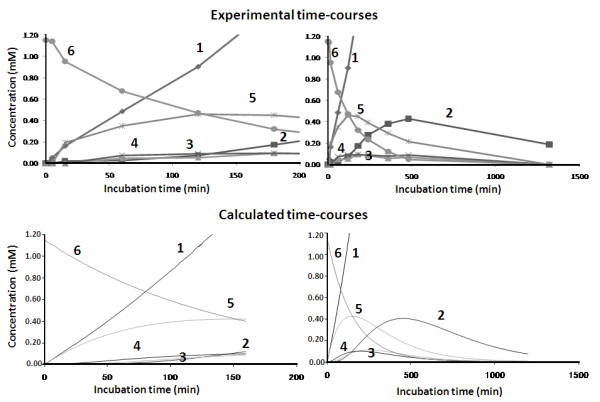
**Time-course of GlcNAc_6 _hydrolysis catalyzed by *Vh*Nag2**. Upper panels: Experimental time-courses obtained by HPLC determination. The early stage of the reaction time-course is shown in the left panel, and the later stage is shown in the right panel. The reaction conditions are described in the text. Lower panels: Calculated time-courses best fitted to the experimental ones. The calculation was conducted using the rate constants and binding free energy values listed in Table 2.

The time course of chitin oligosaccharide hydrolysis by *Vh*Nag2 was further investigated by quantitative HPLC. Fig. 5A shows GlcNAc_2 _hydrolysis, showing a rapid increase of the resultant product (GlcNAc) with increasing reaction time. Fig. 5B and Fig. 5C show GlcNAc_3 _and GlcNAc_4 _hydrolysis. In agreement with the TLC results, GlcNAc_3 _and GlcNAc_4 _were consumed more rapidly than GlcNAc_2 _(Fig. 5A), GlcNAc_5 _(Fig. 5D) and GlcNAc_6 _(Fig. 5E). At the end of recording (180 min), no substrate was left from the digestion of GlcNAc_3 _and GlcNAc_4_, whereas substantial amounts of the other substrates remained. All substrates were hydrolyzed exolytically, so that GlcNAc_n _was degraded to GlcNAc_n-1 _+ GlcNAc. The intermediate products, such as GlcNAc_n-1 _and GlcNAc_n-2_, were further degraded to the final product GlcNAc. Fig. 5F shows the overall rates of hydrolysis to be in the order: GlcNAc_4 _≅ GlcNAc_3 _> GlcNAc_5 _> GlcNAc_6 _> GlcNAc_2_. The substrate-size dependence of the reaction rate can be confirmed from the reaction time-course of extensive hydrolysis of GlcNAc_6 _over a longer period of incubation (1,500 min (25 h), the upper right panel of Fig. 6); that is, chitin pentamer and dimer accumulated during the course of the reaction, whereas tetramer and trimer were generated only transiently before being hydrolyzed instantaneously by the enzyme. Of the natural glycosides, GlcNAc_4 _was found to be the best substrate for *Vh*Nag2. Similar results were reported for *β*-GlcNAcidase (exoI) from *Vibrio furnssii *[27]. This enzyme showed its highest *V*_max _towards GlcNAc_4_, while the *V*_max _values decreased 1.1-fold with GlcNAc_3 _and 1.4-fold with GlcNAc_2_. Further sequence analysis suggested no putative signal peptide in the sequences of *Vh*NAg1 and *Vh*Nag2. Therefore, the two enzymes are suggested to be non-secretory proteins that act intracellularly. In bacterial cells, the transport of chitin oligomers into the intracellular compartments for further degradation by these GlcNAcases could be achieved by specific membrane transporters. It has been proposed for *V. furnissii *that the uptake of short-chain chitooligosaccharides, such as GlcNAc_3 _and GlcNAc_4_, takes place through a chitooligosaccharide-specific channel known as chitoporin [29,30].

### Kinetic modeling and subsite mapping

**Figure 7:**
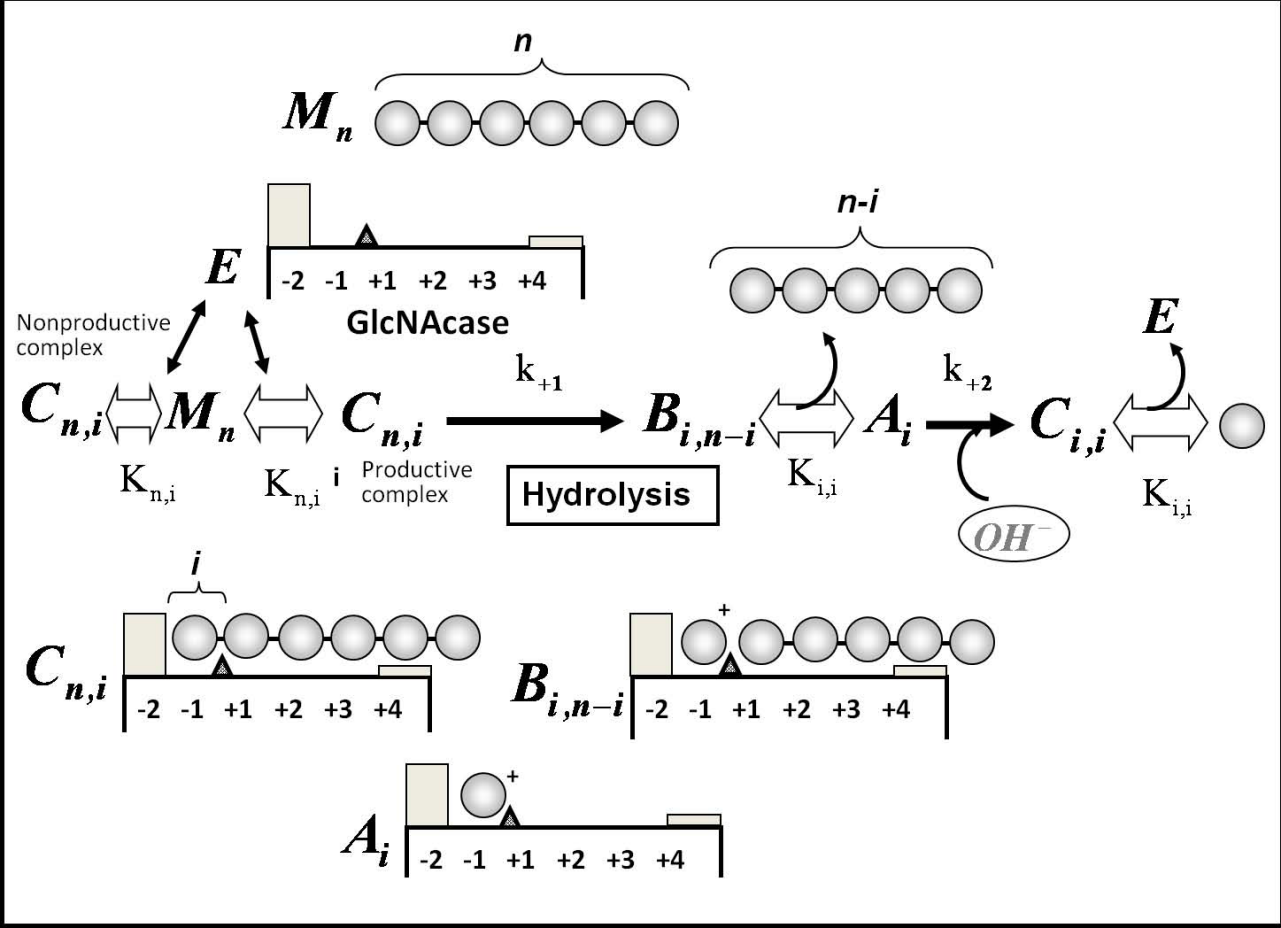
**Kinetic model of the GlcNAc_6 _hydrolysis catalyzed by *Vh*Nag2**. *M*_n _and *E *represent free oligosaccharide substrate and the free enzyme, respectively. The subscripts *n *and *i *indicate the size of GlcNAc oligosaccharide. *A*_*i*_*, B*_*i,n-i*_, and *C*_*n,i *_represent the enzyme-substrate complexes at various stages as shown in the figure. The gray-boxes indicate the subsites possessing unfavorable positive binding free energy changes (-2 and +4 subsites). At subsite +4, the sugar residue binding is moderately unfavorable, but at subsite -2 sugar residue binding is strictly prohibited. The simultaneous differential equations were derived from the reaction model, and numerically solved to obtain the calculated time-course. The details of the calculation method are described in ref. [31].

Kinetic modeling of the enzymic reaction was carried out based on the reaction scheme presented in Fig. 7. To model the time-course of GlcNAc_6 _hydrolysis (shown in the upper panels of Fig. 6), numerical calculations were conducted using the differential equation derived from the reaction scheme [31]. At first, the binding free energy changes for the subsites from (-1) to (+4) were estimated roughly by repeating the calculations with various values of the free energy changes. Starting with initial guessed values, optimization of the free energy changes conducted by the modified Powell method [32] was employed using the cost function,

(1) $$F=\sum_{i}\sum_{n}{\left[(\text{GlcNAc})c_{n,i}-(\text{GlcNAc})e_{n,i}\right]}^{2}$$

where *e *and *c *represent the experimental and calculated values, respectively, *n *is the number of GlcNAc units in the oligosaccharides, and *i *the reaction time.

The final iteration of the calculations yielded the values of the rate constants for glycosidic bond cleavage (*k*_+1_) for various chain lengths of GlcNAc_n_. The *k*_cat _values obtained were allocated to *k*_+1 _for individual oligosaccharide substrates (Table 1). The individual *k*_+1 _values were fixed in the modeling calculation. Since no evidence for transglycosylation was obtained from HPLC determination of the enzymic products, this process was not considered in the kinetic model. A much higher value of 50 s^-1 ^was tentatively allocated to the rate constant for hydration *k*_+2_. Since *Vh*Nag2 splits off a monosaccharide from the non-reducing end of the substrate, indicating that subsite (-2) should be the most unfavorable for binding of the GlcNAc residue, we tentatively allocated a high positive value (+7.0 kcal/mol) to the binding free energy change at subsite (-2). Using the fixed values of *k*_+1_, *k*_+2_, and the binding free energy change of subsite (-2), the values of binding free energies of individual subsites from (-1) to (+4) were optimized on the basis of the experimental time-course (upper panels of Fig. 6). As shown in lower panels of Fig. 6, the calculated time-courses were satisfactorily fitted to the experimental ones, for both the early stage (left panels) and the later stage (right panels) of the reaction, yielding the binding free energy changes of the individual subsites as listed in Table 2.

**Table 2:** The rate constants and the binding free energy changes estimated from the kinetic modeling calculation.

Substrate	**Rate constant (s**^**-1**^**)**			**Binding free energy (*k*cal mol**^**-1**^**)**					
	
	***k***_**+1**_	***k***_**-1**_	***k***_**+2**_	(-2)	(-1)	(+1)	(+2)	(+3)	(+4)
GlcNAc_4-6_	0.07-0.1	0.0	50.0	+7.0	-4.5	0.0	-1.1	-0.1	+0.9

The optimization was based on the experimental time-course of hexamer hydrolysis catalyzed VhNag2 (upper panels of Fig. 6).

The TLC and kinetic data suggested that the catalytic pocket of *Vh*Nag2 probably contains a sequence of four favorable GlcNAc binding subsites, designated (-1)(+1)(+2)(+3). Such an implication is certainly supported by the binding free energy changes obtained by the kinetic modeling. As shown in Table 2, a very high positive value +7.0 kcal/mol for subsite (-2) suggests that there is a large steric hindrance interfering with binding at this subsite. A less positive value of +0.9 kcal/mol of subsite (+4) suggests that the sugar residue binding to this subsite is moderately unfavorable, but still possible. Thus, GlcNAc_4 _binds to (-1) (+1), (+2), and (+3) more strongly than GlcNAc_5 _to (-1) (+1), (+2), (+3) and (+4). A low negative free energy change, -0.1 kcal/mol, was estimated for binding at subsite (+3). This is consistent with the fact that the GlcNAc_3 _binding affinity to the subsites (-1) (+1), and (+2) (*K*_m _= 441 μM) is somewhat weaker than that of the GlcNAc_4 _binding to (-1) (+1), (+2), and (+3) (*K*_m _= 329 μM). All of these results clearly indicate that an array of four GlcNAc binding subsites (-1)(+1)(+2)(+3) define the substrate affinity of *Vh*Nag2. It appears that the GlcNAcases reported to date possess two major types of substrate specificity. The first type has marked preference towards chitobiose, while the other type favours chitooligomers (GlcNAc_3-6_) over chitodimer. Examples of the former type are *hyperthermophilic kodakaraensis *KOD1 GlmA_TK _[33], *Sm*Chb [25], and *S. thermoviolacacus *NagC [28]. On the other hand, *V. furnissii *exoI [27], human di-*N*-acetylchitobiase [34], and *Vh*Nag2 (in this report) are among the other type. The active site of the second type of GlcNAcases has been demonstrated to contain three to five GlcNAc binding subsites, depending on the substrate specificity of individual enzymes. Multiple sugar-binding-site architecture is not uncommon, as has been demonstrated for other exo-glycosidases such as *Aspergillus niger *cellobiase [35], a GH-3 enzyme that degrades cellobioase and cello-oligosaccharides into glucose units. The catalytic center of this enzyme has been reported to contain up to five binding subsites. Also, structural studies of the active site of *Bacillus halodurans *C-125 REX [36], a GH-8 exo-oligoxylanase that hydrolyzes xylooligosaccharides to xylose from the reducing end, revealed three substrate binding subsites.

## Conclusions

This study reports the isolation, cloning and recombinant expression of the genes encoding two intracellular GH-20 GlcNAcases from a marine bacterium, *Vibrio harveyi *type strain 650. Data obtained from TLC and quantitative HPLC suggested that the active GlcNAcase homolog (*Vh*Nag2) was an exolytic enzyme that degraded chitin oliogmers, releasing GlcNAc as the end product. Kinetic modeling suggested that the active site of *Vh*Nag2 comprises four GlcNAc binding subsites, (-1), (+1), (+2), (+3). Such subsite identification is strongly supported by kinetic data, which showed chitin tetramer as the most effective substrate for this enzyme.

## Methods

### Bacterial strains and vectors

*V. harveyi *type strain 650 was a marine isolate from Greek sea bass and was a gift from Professor Brian Austin, Heriot-Watt University, Edinburgh, United Kingdom. *E. coli *strain DH5*α *was used for routine cloning and plasmid preparations. pGEM^®^-T easy vector used for subcloning purpose was a product of Promega (Promega Pte Ltd, Singapore Science Park I, Singapore). The pQE 60 expression vector and *E. coli *type strain M15 (Qiagen, Valencia, CA, USA) were used for cloning and a high-level expression of recombinant GlcNAcases.

### Cloning of the DNAs encoding VhNag1 and VhNag2

Three sets of oligonucleotide primers were designed based on the three putative GlcNAce genes, designated VIBHAR_01265, VIBHAR_03430, VIBHAR_06345) from *V. harveyi *type strain ATCC BAA-1116 in the CAZy database. However, only two PCR products, namely *VhNag1 *and *VhNag2*, were successfully amplified from the genomic DNA of *V. harveyi *type strain 650. The oligonucleotides used for amplification of *VhNag1 *DNA are 5'-AGGATCCGGGCAGGGTAAAATC-3' for the forward primer and 5'-AGGAGATCTATCGGTTAAAGTGTGAAG-3' for the reverse primer. For *VhNag2 *DNA, 5'-AGGGATCCGAATACCGTGTTGATTTA-3' was used as the forward primer and 5'-AATAGATCTCTTCCACGGTTTACGGTA-3' for the reverse primer. The PCR products of expected sizes (2.3 kbp for *VhNag1 *and 1.9 kbp for *VhNag2*) were cloned in the pQE60 expression vector using *Bam*H I and *Bgl *II cloning sites (sequences underlined) following the protocol supplied by the manufacturer.

### Nucleotide, amino acid sequence and phylogenic analyzes

The nucleotide sequences of *VhNAg1 *and *VhNag2 *were determined by automated double stranded DNA sequencing (Bio Service Unit, Thailand Science Park, Bangkok, Thailand). Ambiguous nucleotides were re-confirmed twice before submission to the Genbank database. The amino acid sequence alignment was constructed using ''CLUSTALW'' algorithm commercially available in Lasergene v.7 (DNASTAR, Inc., WI, USA) and displayed using the Genedoc program (http://www.psc.edu/biomed/genedoc/). The putative sequences of VhNag1 and VhNag2 were aligned with the previously published *V. harveyi *chitobiase [26] together with four bacterial and two human GlcNAcases of known structures.

### Protein expression and purification

The full-length *Vh*Nag1 and *VhNag2 *DNAs were cloned into pQE60 expression vector and expressed in *E. coli *M15 host as the *C*-terminally (His)_6_-tagged polypeptides. The cells were grown at 37°C in Luria Bertani (LB) medium containing 100 μg/ml ampicillin until the OD_600 _of the cell culture reached 0.6. Expression was induced by the addition of isopropyl thio-*β*-D-galactoside (IPTG) to a final concentration of 0.5 mM. After 18 h of induction at 20°C, the cell pellet was collected by centrifugation, re-suspended in lysis buffer (20 mM Tris-HCl buffer, pH 8.0, containing 150 mM NaCl, 1 mM phenylmethylsulphonyl fluoride (PMSF), and 1.0 μg/ml lysozyme), and then lysed on ice using a Sonopuls Ultrasonichomogenizer with a 6-mm-diameter probe (50% duty cycle; amplitude setting, 20%; total time, 30 s, 6-8 times). Unbroken cells were removed by centrifugation at 12,000 × g, 20 min at 4^ο^C. The supernatant was immediately applied to a Ni-NTA agarose affinity column (1 × 10 cm) (QIAGEN GmbH, Hilden, Germany), and the chromatography was carried out under gravity at 4°C. The column was washed thoroughly with 5 mM imidazole, followed by 20 mM imidazole in equilibration buffer (20 mM Tris-HCl buffer, pH 8.0), and then 250 mM imidazole in the same buffer. Three eluted fractions (10 ml each) were collected and analyzed by 12% SDS-PAGE [37] to confirm purity. GlcNAcase fractions were pooled and then subjected to several rounds of membrane centrifugation using Vivaspin-20 ultrafiltration membrane concentrators (*M*_r _10,000 cut-off, Vivascience AG, Hannover, Germany) for complete removal of imidazole. The final concentration of the protein was determined by Bradford's method [38].

### Confirmation of recombinant expression by mass spectrometry

The purified *Vh*Nag1 and *VhNag2 *(2 μg) were applied in parallel onto a 12% SDS-PAGE gel, and stained with Coomassie blue R-250 after electrophoresis. After destaining, protein bands were subjected to in-gel digestion with trypsin (sequencing grade, Promega) using a standard protocol [39]. The resultant peptides were analyzed by high resolution nanoESI/FTMS by the mass spectrometry facility located at the Max-Planck Institute for Molecular Physiology, Dortmund. Data bank searching was performed with ''Mascot search'' (http://www.matrixscience.com/) for peptide mass fingerprinting.

### GlcNAcase activity assays

GlcNAcase activity was determined spectrophotometrically using *p*NP-GlcNAc (Sigma-Aldrich Pte Ltd., The Capricorn, Singapore Science Park II, Singapore) as substrate or by a reducing sugar assay using GlcNAc_2-6 _(AMS Biotechnology (Europe) Ltd, Oxfordshire, UK) and colloidal chitin as substrates. For the *p*NP assay, a 100-μl assay mixture contained the protein sample (50 μg), 125 μM *p*NP-GlcNAc), and 0.065 M phosphate buffer, pH 7.0 The enzymic reaction was continued for 10 min at 37°C before being terminated by the addition of 100 μl 3 M Na_2_CO_3_. The amount of *p*-nitrophenol (*p*NP) released was determined spectrophotometrically at 405 nm in a microtiter plate reader (Applied Biosystems, Foster City, CA, USA). Molar concentrations of *p*NP were calculated from a calibration curve constructed with 0-20 nmol *p*NP. For the reducing sugar assay, the reaction mixture (100 μl) contained 250 μM GlcNAc_2-6 _in 0.1 M phosphate buffer, pH 7.0 and 200 μg enzyme or 500 μM *p*NP-glycoside in 0.1 M phosphate buffer, pH 7.0 and 100 μg enzyme. The reaction mixture was incubated at 37°C for 15 min in a Thermomixer comfort (Eppendorf AG, Hamburg, Germany), then heated at 100°C for 10 min. The entire reaction mixture was subjected to 3,5-dinitrosalicylic acid (DNS) assay following the protocol described by Miller [40]. Release of the reducing sugars was detected spectrophotometrically at 540 nm and molar concentrations of the released sugars were estimated using a standard calibration curve of GlcNAc (0-500 nmol). For colloidal chitin, the reaction mixture (200 μl), containing 5% (w/v) colloidal chitin (prepared according to Hsu and Lockwood, 1975 [41]), 0.1 M phosphate buffer, pH 7.0, and 200 μg enzyme, was incubated at 37°C for 15 min. After centrifugation at 12,000 × *g *for 1 min to precipitate the remaining chitin, the product concentration in the supernatant (100 μl) was determined by DNS method as described for GlcNAc_2-6_.

### Effects of pH on the enzymatic activity

A discontinuous assay was used to determine the pH profiles of *Vh*Nag1 and *Vh*Nag2. The reaction mixtures containing 500 μM *p*NP-GlcNAc were pre-incubated at 37°C for 5 min at different pH values ranging from 2.5 to 9.0 using the McIlvaine's sodium phosphate-citric acid - KCl buffer system [42], followed by addition of 1 μg *Vh*Nag1 or 0.5 μg *Vh*Nag2. After 10 min of incubation, the reactions were terminated with 100 μl of 3 M Na_2_CO_3_. The amounts of *p*NP released were estimated as described for the *p*NP assay.

### Time course of substrate analysis by thin-layer chromatography

Hydrolysis of chitooligosaccharides (GlcNAc_2-6_) by *Vh*Nag2 was carried out in a 20-μl reaction mixture, containing 0.1 M phosphate buffer, pH 7.0, 2.5 mM substrate and 5 μg purified enzyme. The reaction mixture was incubated at 30°C for 1, 5, 10, 15, 30 min, 3 h or 18 h, and the reaction terminated by boiling for 5 min. For product analysis, five 1-μl aliquots of each reaction mixture were applied to a silica TLC plate (7 × 10 cm), and then chromatographed three times (1 h each) in a mobile phase containing *n*-butanol:methanol:28% ammonia solution:H_2_O (10:8:4:2) (v/v), followed by spraying with aniline-diphenylamine reagent and baking at 180°C for 3 min. To determine the time course of chitin hydrolysis, the reaction was carried out in a 150-μl reaction mixture, containing 0.1 M phosphate buffer, pH 7.0, 20 mg colloidal chitin, and 50 μg purified enzyme. Subsequent reactions and determination of the reaction products were analyzed by TLC as described for chitooligosaccharide hydrolysis.

### Time-course analysis of chitooligosaccharide hydrolysis by HPLC

A reaction mixture (100 μl) containing 1.25 mM chitin oligosaccharide (GlcNAc_2-6_), 38 μM *Vh*Nag2 and 0.2 M sodium phosphate buffer, pH 7.0 was incubated at 30°C. An aliquot of 12 μl was transferred to a new microfuge tube containing 12 μl 0.1 M NaOH after 5, 10, 15, 30, 60, 120 and 180 min, and the enzymic reaction was stopped by snap-freezing in liquid N_2 _and the mixture immediately stored at -20°C. To quantitatively determine the time-course of substrate degradation and product formation, 15-μl of the reaction mixture was applied to a gel-filtration column of TSK-GEL G2000PW (7.5 × 600 mm, Tosoh) connected with a Hitachi L-7000 HPLC system (Hitachi Koki Co., Ltd, Tokyo). Elution was conducted with distilled water at a flow rate of 0.3 ml/min, and the substrate and products were monitored by their absorption at 220 nm. Based on the peak areas obtained from the elution profiles, oligosaccharide concentrations were calculated using a standard curve obtained with authentic saccharide solutions, and then plotted against the reaction time to obtain the reaction time-course.

### Steady-state kinetics

Kinetic parameters were determined using *p*NP-GlcNAc and chitooligosaccharides (GlcNAc_2-6_) by the reducing sugar assay as described above, with 0-500 μM of each substrate in the reaction mixture. The amounts of the reaction products were determined from a standard curve of GlcNAc (0-1.75 μmol). Kinetic parameter values were evaluated from three independent sets of data using the nonlinear regression function obtained from the GraphPad Prism v.5.0 (GraphPad Software Inc., San Diego, CA).

### Kinetic modeling of substrate hydrolysis

Kinetic modeling of the reaction time-course obtained by HPLC was carried out using the reaction model reported for the *Coccidioides immitis *family 18 chitinase [43]. The model scheme is shown in Fig. 7. Considering that the enzyme hydrolyzes the oligosaccharide substrate exolytically from the non-reducing end, *Vh*Nag2 was assumed to have a (-2)(-1)(+1)(+2)(+3)(+4)-type binding cleft, where subsite (-2) should have an unfavorable (positive) binding free energy change. By assuming rapid binding equilibrium, the concentrations of the ES-complexes formed through the individual binding modes (C_*n,i*_, B_*i,j*_, and A_*i*_) were calculated from the binding constants, which were obtained from the binding free energy values of individual subsites occupied with the sugar residues assuming additivity. Details of the calculation method were described by Honda and Fukamizo [31].

## Abbreviations

GlcNAc_n _or NAG: *β*1-4 linked oligomers of *N*-acetylglucosamine residues where n = 1-6; GlcNAcase: *β *-*N*-acetyl-glucosaminidase; DNS: 3,5-dinitrosalicylic acid; *p*NP-GlcNAc: *p*-nitrophenyl-*β*-D-*N*-acetylglucosaminide; IPTG: isopropyl thio-*β*-D-galactoside; ORFs: open reading frames; PMSF: phenylmethylsulphonylfluoride; TLC: Thin Layer Chromatography.

## Authors' contributions

WS initiated the research, was responsible for the theme setting, primer design and site-directed mutagenesis. She also carried out HPLC experiments, performed analysis and interpretation of the kinetic, TLC and HPLC data, and also prepared and finalized the manuscript. DC carried out genomic DNA isolation, cloning, recombinant expression, protein purification, TLC and kinetic experiments. MM performed the calculation of the rate constants and binding free energy changes. TF provided guidance on quantitative HPLC, theoretical analysis and deduction of the reaction scheme. He also edited and proofread the manuscript. All authors read and approved the final manuscript.

## Supplementary Material

Additional file 1 **Table S1 Identification of *V. harveyi *GlcNAcases by mass spectrometry**. Tryptic peptides of *Vh*Nag1 and *Vh*NAg2 were resolved and detected by nano-HPLC/ESI-FTMS. Mascot search subsequently identified the resultant peptides of *β*-N-acetyl glucosaminidases from the NCBINr database. Click here for file

Additional file 2 **Fig. S1 The reaction progress curve of *Vh*Nag1 and *Vh*Nag2 using *p*NP-GlCNAc as substrate**. The reaction mixtures (200 μl) containing 250 μM *p*NP-GlcNAc, 10 μg *Vh*Nag1 or 5 μg *Vh*Nag2, and 0.1 M sodium phosphate buffer, pH 7.0 were incubated at 37°C for 5, 10, 15, 30, 45, 60 and 180 min. After the specified time the reaction was terminated by the addition of 100 μl 3 M Na_2_CO_3_. The release of *p*NP was determined as described in the main text. Click here for file
