# Supplementary material for: Novel β-N-acetylglucosaminidases from Vibrio harveyi 650: Cloning, expression, enzymatic properties, and subsite identification
Source: BMC Biochem. 2010 Sep 29;11:40. doi: 10.1186/1471-2091-11-40 (PMC2955587; doi:10.1186/1471-2091-11-40)
Supplement: Additional file 1 — Table S1 Identification of V. harveyi GlcNAcases by mass spectrometry. Tryptic peptides of VhNag1 and VhNAg2 were resolved and detected by nano-HPLC/ESI-FTMS. Mascot search subsequently identified the resultant peptides of β-N-acetyl glucosaminidases from the NCBINr database. [file 1471-2091-11-40-S1.DOC]

Supplementary Table S1

| Observed  monoisotopic mass (MH^+^) | Charge (Z) | Peptide sequence | Protein ID | Identified protein |
| --- | --- | --- | --- | --- |
| *Vh*Nag1 |  |  |  |  |
| 442.7084  1055.0401 | 2  2 | R.GVSYDTSR.H  K.DLPTSIVIQSWQGHDSIGR.A | gi|  84386131 | N-acetyl-β-hexosaminidase  [*Vibrio splendidus* 12B01] |
| 429.2415  442.7084  458.2134  512.2765  579.7795  627.8628  434.8691  841.9339  946.1122 | 2  2  2  2  2  2  3  2  3 | K.SFSIYIK.G  R.GVSYDTSR.H  R.AFTDYNGK.S  R.GLQSYLNTK.V  R.AFTDYNGKSR.E  R.HFIELDVILR.Q  K.MTGWDEIWHK.D  K.SIVIQSWQGHDSIGR.A  K.EGYQGILSTGYYLDQPQPTSYHYR.N | gi|  90578682 | N-acetyl-β-hexosaminidase  [*Vibrio angustum* S14] |
| 353.6976  388.7367  388.7367  429.2415  431.7478  442.7084  458.2134  512.2765  383.8657  579.7795  627.8628  434.8691  841.9339 | 2  2  2  2  2  2  2  2  3  2  2  3  2 | R.VQFNAK.R  K.IQQFIK.D  R.SYAVAER.L  K.SFSIYIK.G  R.VQFNAKR.T  R.GVSYDTSR.H  R.AFTDYNGK.S  R.GLQSYLNTK.V  R.HHADANIMLK.R  R.AFTDYNGKSR.E  R.HFIELDVILR.Q  K.MTGWDEIWHK.D  K.SIVIQSWQGHDSIGR.A | gi|  89074271 | N-acetyl-β-hexosaminidase [*Photobacterium sp.* SKA34] |
| *Vh*Nag2 |  |  |  |  |
| 434.7350  477.2030  500.2713  528.2427  555.7875  565.3261  582.7828  422.8950  432.8909  492.9035  797.9385  814.9538  563.2973  612.6872  638.6863  966.9709  1031.0179  1049.4992  1102.5731  804.7335  1317.6454 | 2  2  2  2  2  2  2  3  3  3  2  2  3  3  3  2  2  2  2  3  2 | R.HAEDKLR.K  R.GMMLDCAR.H  K.ELQGHFLR.H  R.DWTDYLSR.L  R.HFHSVEQVK.R  R.VDLVVLSEQK.Q  K.GNPTLDEGAYK.L  R.HFHSVEQVKR.L  R.DWTDYLSRLK.G  K.QHRDWTDYLSR.L  K.GHLPLLDLQGVNYR.N  R.AQVDVTPIVLASPYR.E  R.VDLVVLSEQKQNCR.F  R.LKGHLPLLDLQGVNYR.N  R.AQVDVTPIVLASPYRER.S  K.AYNYEPLAEVPADDPIR.K  K.AYNYEPLAEVPADDPIRK.R  K.DTVIYSWLSEEAALNCAR.Q  K.IEAGSSSGFTHACATLLQLIK.V  K.VSKDTVIYSWLSEEAALNCAR.Q  R.SIQHYNDNVINPALPGSYEFIDK.V | gi|  799237 | β-N-hexosaminidase [*Vibrio parahaemolyticus*] |
| 477.2030  528.2427  536.7506  565.3261  582.7828  432.8909  492.9035  797.9385  540.6236  814.9538  838.9433  563.2973  612.6872  638.6863  966.9709  1031.0179  1049.4992  1102.5731  804.7335  1317.6454 | 2  2  2  2  2  3  3  2  3  2  2  3  3  3  2  2  2  2  3  2 | R.GMMLDCAR.H  R.DWTDYLSR.L  R.MDYMIFPR.L  R.VDLVVLSEQK.Q  K.GNPTLDEGAYK.L  R.DWTDYLSRLK.G  K.QHRDWTDYLSR.L  K.GHLPLLDLQGVNYR.K  R.GITIIPEIDVPGHCR.A  R.AQVDVTPIVLASPYR.E  R.GITIIPEIDVPGHCR.A  R.VDLVVLSEQKQNCR.F  R.LKGHLPLLDLQGVNYR.K  R.AQVDVTPIVLASPYRER.S  K.AYNYEPLAEVPADDPIR.K  K.AYNYEPLAEVPADDPIRK.R  K.DTVIYSWLSEEAALNCAR.Q  K.IEAGSSSGFTHACATLLQLIK.V  K.VSKDTVIYSWLSEEAALNCAR.Q  R.SIQHYNDNVINPALPGSYEFIDK.V | gi|  91228309 | β-N-hexosaminidase [*Vibrio alginolyticus* 12G01] |
| 477.2030  685.8747  704.6731 | 2  2  3 | R.GMMLDCAR.H  K.SLPQLTEIGAWR.G  K.ETIIYSWLSEEAAVNCAR.Q | gi|  84393823 | N-acetyl-β-hexosaminidase [*Vibrio splendidus* 12B01] |
