# Supplementary figures and images for: Novel β-N-acetylglucosaminidases from Vibrio harveyi 650: Cloning, expression, enzymatic properties, and subsite identification
Source: BMC Biochem. 2010 Sep 29;11:40. doi: 10.1186/1471-2091-11-40 (PMC2955587; doi:10.1186/1471-2091-11-40)

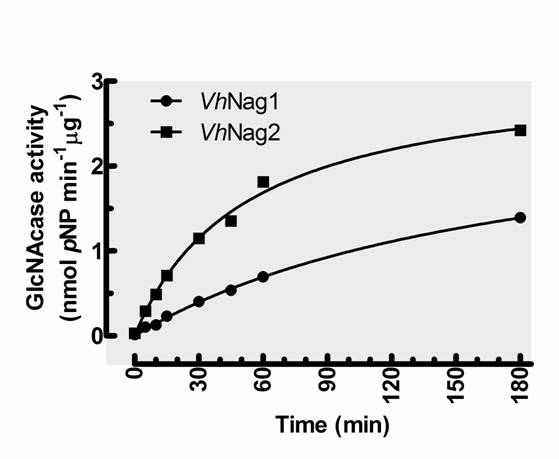

Supplement: Additional file 2 — Fig. S1 The reaction progress curve of VhNag1 and VhNag2 using pNP-GlCNAc as substrate. The reaction mixtures (200 μl) containing 250 μM pNP-GlcNAc, 10 μg VhNag1 or 5 μg VhNag2, and 0.1 M sodium phosphate buffer, pH 7.0 were incubated at 37°C for 5, 10, 15, 30, 45, 60 and 180 min. After the specified time the reaction was terminated by the addition of 100 μl 3 M Na2CO3. The release of pNP was determined as described in the main text. [file 1471-2091-11-40-S2.TIFF]
